# Supplementary material for: Causal Relationship between Aging and Anorexia Nervosa: A White-Matter-Microstructure-Mediated Mendelian Randomization Analysis
Source: Biomedicines. 2024 Aug 16;12(8):1874. doi: 10.3390/biomedicines12081874 (PMC11351342; doi:10.3390/biomedicines12081874)
Supplement: Supplementary file 1 [file biomedicines-12-01874-s001.zip › Supplementary Figure/Figure S9/Leaveoneout plot of MR analysis for ubm-b-1466 to AN.pdf]

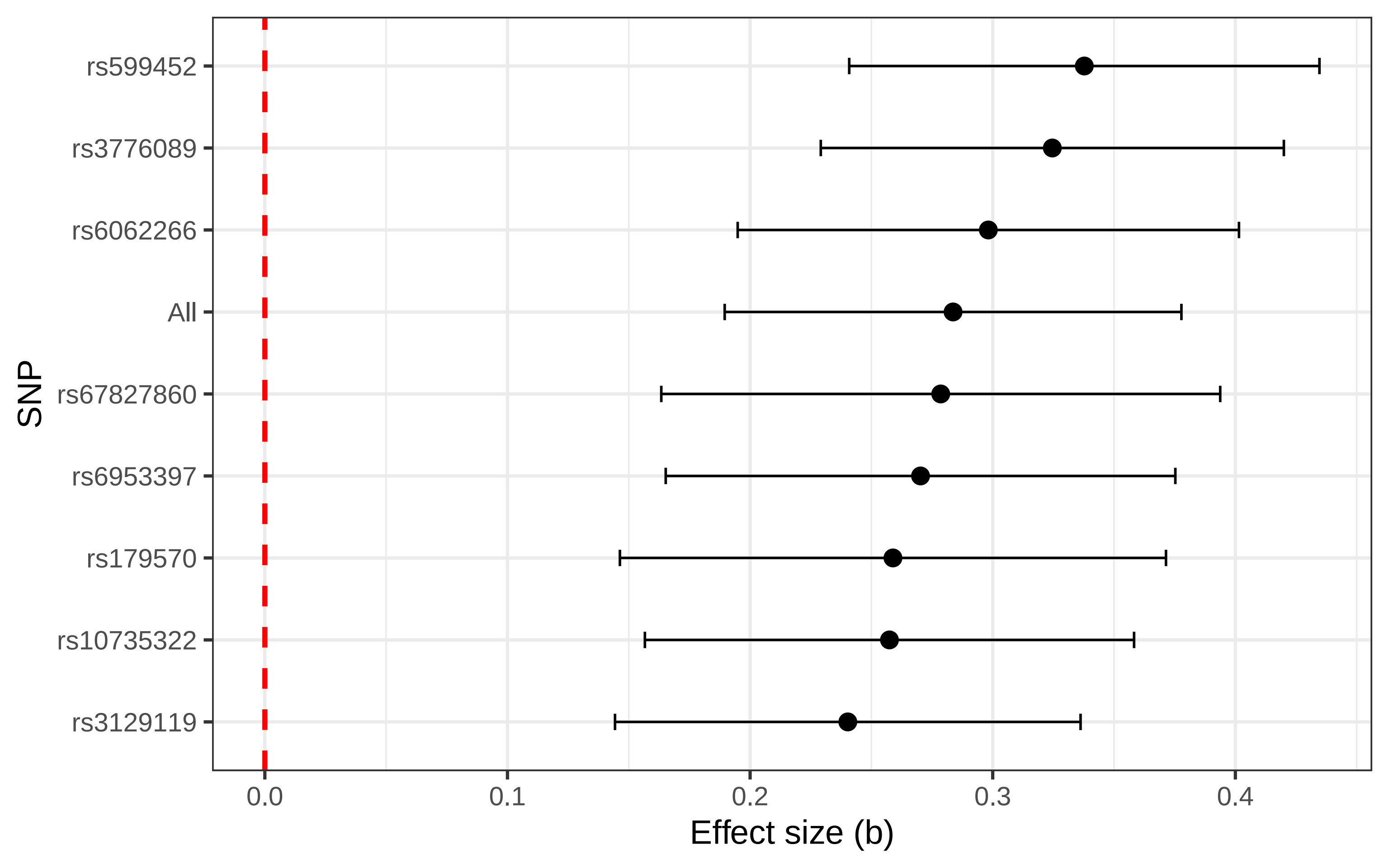

rs599452
rs3776089
rs6062266
All
rs67827860
rs6953397
rs179570
rs10735322
rs3129119
SNP
0.0
0.1
0.2
0.3
0.4
Effect size (b)
